# Effects of sex and obesity on immune checkpoint inhibition-related cardiac systolic dysfunction in aged mice

**DOI:** 10.1007/s00395-024-01088-4

**Published:** 2024-11-08

**Authors:** Nabil V. Sayour, Dániel Kucsera, Ayham R. Alhaddad, Viktória É. Tóth, Tamás G. Gergely, Tamás Kovács, Zsombor I. Hegedűs, Márk E. Jakab, Péter Ferdinandy, Zoltán V. Varga

**Affiliations:** 1 Department of Pharmacology and Pharmacotherapy, Semmelweis University, Budapest, Hungary; 2 Center for Pharmacology and Drug Research & Development, Semmelweis University, Budapest, Hungary; 3 HCEMM-SE Cardiometabolic Immunology Research Group, Semmelweis University, Budapest, Hungary; 4 MTA-SE Momentum Cardio-Oncology and Cardioimmunology Research Group, Semmelweis University, Budapest, Hungary; 5 Pharmahungary Group, Szeged, Hungary

**Keywords:** Immune checkpoint inhibitor, Cardiotoxicity, Obesity, Hypercholesterolemia, Sex, T-cell exhaustion

## Abstract

Despite accumulating data on underlying mechanisms, the influence of sex and prevalent cardio-metabolic co-morbidities on the manifestation and severity of immune checkpoint inhibitor (ICI)-induced cardiotoxicity has not been well defined. To elucidate whether sex and prevalent cardio-metabolic co-morbidities affect ICI-induced cardiotoxicity, we randomized 17-month-old male and female mice to receive control diet (CON) or high-fat diet (HFD) + L-NAME—a well-established mouse model of cardio-metabolic co-morbidities—for 17 weeks (*n* = 5–7), and evaluated markers of T-cell function in the spleen. As expected, HFD + L-NAME significantly increased body- and heart weight, and serum cholesterol levels, and caused no systolic dysfunction, however, led to diastolic dysfunction, cardiomyocyte hypertrophy, and increased fibrosis only in males compared to corresponding CON. Western blot analyses of splenic immune checkpoint protein levels showed differential expression depending on sex and prevalent cardio-metabolic co-morbidities, suggesting T-cell exhaustion in both sexes on HFD + L-NAME, but more pronounced in males. In a sub-study with a similar setup, we tested cardiotoxic manifestations of ICI by treating mice with anti-PD-1 monoclonal antibody (ICI) for the last 2 weeks of diet administration (*n* = 5–7). After 2 weeks of ICI treatment, cardiac systolic functions significantly decreased in CON, but not in HFD + L-NAME groups of both sexes compared to baseline (before ICI administration). In conclusion, in this exploratory study using aged mice, we describe for the first time that ICI-related systolic dysfunction is diminished in both sexes when obesity and hypercholesterolemia are present, possibly due to obesity-related T-cell exhaustion.

**Supplementary Information:**

The online version contains supplementary material available at 10.1007/s00395-024-01088-4.

## Introduction

Immune checkpoint molecules exert inhibitory or stimulatory effects on immune responses, and thus, are pivotal for regulating immune functions in both physiologic and pathophysiologic conditions. In physiological conditions, immune checkpoint molecules are crucial to maintaining self-tolerance, whereas in pathophysiological states (e.g., in cancer or chronic infection), these molecules mediate immune invasion, and are key for maintaining effector functions of immune cells, more specifically, of T cells [12]. However, when inflammation remains persistent, T-cell differentiation becomes altered, manifesting as loss of effector functions, sustained upregulation of inhibitory receptors, and altered expression of key transcription factors, overall resulting in T-cell exhaustion [54].

In cancer, overexpression of inhibitory immune checkpoint molecules may often occur, leading to T-cell exhaustion that prevents optimal anti-tumor defense mechanisms. Therefore, modulating this pathway can reverse this dysfunctional state and reinvigorate effective anti-tumor immune responses. The most effective modifiers for these molecules are the immune checkpoint inhibitors (ICIs) that have revolutionized cancer therapy by increasing anti-tumor responses of the immune cells and are being used and tested in a large array of cancer types showing almost unanimous benefit for cancer patients [59].

Nevertheless, cardiotoxic side effects of ICIs, beyond myocarditis and pericarditis [44, 46], have been increasingly recognized, ranging from systolic dysfunction to cardiac dysrhythmias [7]. As the number of cancer patients eligible for ICI therapy is rapidly increasing [15], the number of patients at risk for ICI-related cardiac side effects is also rising at a similar pace. Therefore, understanding the determinants and the mechanism of ICI-related cardiotoxicities is of paramount importance to develop preventive, or curative strategies against these harmful side effects, without hampering anti-cancer efficacy.

Although conditions associated with increased or decreased anti-cancer efficacy of ICIs have become well-established over the past years [31], currently, conditions that may be associated with an increased or decreased risk for ICI-induced cardiotoxicities remain to be further investigated, as currently, evidence is scarce. For instance, it has been shown that the prevalence of diabetes mellitus or obesity is higher among patients in whom ICI-induced myocarditis occurred [32]. Moreover, data on the effect of sex on immune-related adverse events for ICI treatment are conflicting, with some studies showing no difference between sexes in terms of myocarditis [20], while others show more harm in the female sex [9, 61]. Nevertheless, in general, studies assessing determinants of ICI-related cardiotoxicities other than myocarditis, e.g., systolic dysfunction, are lacking.

In this exploratory in vivo study, we aimed to investigate the effects of ICI treatment on systolic function in the presence or absence of obesity and hypercholesterolemia in aged mice of both sexes. Aged mice were used, as cancer is a disease mostly of people of advanced age. We sought to characterize the general T-cell state in obese, hypercholesterolemic, aged mice of both sexes by measuring protein levels of markers for T-cell exhaustion in the spleen, the largest immune organ with a direct connection to the systemic circulation. Then, we hypothesized how ICI treatment would exert cardiotoxic effects in the same mouse model. We performed all experiments in a randomized and blinded manner.

## Materials and methods

### Ethical approval and animals

All experimental procedures were done in accordance with the Guide for Care and Use of Laboratory Animals published by the US National Institutes of Health (NIH publication No. 85-23, revised 1996), with the EU Directive (2010/63/EU), and in compliance with the ARRIVE 2.0 guidelines, and was approved by the National Scientific Ethical Committee on Animal Experimentation (PE/EA/1912-7/2017, Budapest, Hungary).

Male (*n* = 25) and female (*n* = 25), healthy C57Bl/6 J mice (8 weeks of age) were obtained from the Oncological Research Center, Department of Experimental Pharmacology (Budapest, Hungary), and grew to the age of 17 months, when dietary intervention started. Animals were maintained under 12–12 light–dark cycles under a controlled environment (20–24 °C and 35–75% relative humidity) in individually ventilated cages, holding 2–4 mice per cage. Standard rodent chow and tap water were available ad libitum.

### Study design

**Fig. 1:**
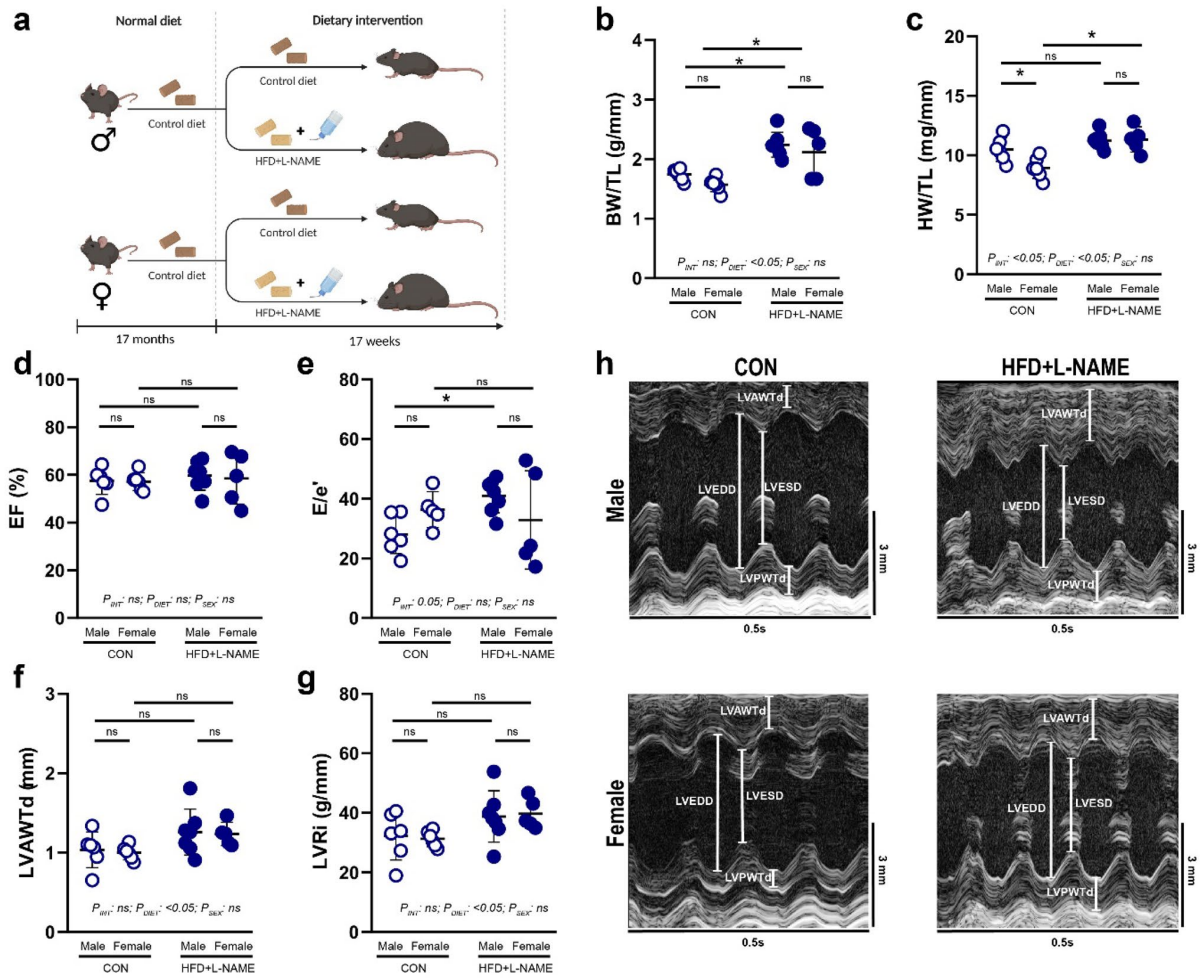
Study design, gross data and echocardiographic measurements of aged mice fed with CON vs HFD + L-NAME. **a** Study design depicting that male and female mice of 17 months of age were randomized into control diet (CON, *n* = 6 for males and *n* = 6 for females) or high fat diet plus L-NAME (HFD + L-NAME, *n* = 7 for males and *n* = 5 for females). **b** Body weight (BW) normalized to tibia length (TL) is significantly higher in the HFD + L-NAME vs. CON groups, irrespective of sex. **c** Heart weight (HW) normalized to TL is significantly higher in the HFD + L-NAME vs. CON groups, irrespective of sex. **d** Left ventricular ejection fraction (EF) is not affected by the diet or sex. **e** *E*/*e*’ ratio, a major determinant for diastolic dysfunction is increased in males, but not in females of the HFD + L-NAME-fed groups. **f** Left ventricular anterior wall thickness in diastole (LVAWTd) is significantly higher in the HFD + L-NAME vs. CON groups, irrespective of sex. **g** Left ventricular remodeling index (LVRi) is significantly higher in the HFD + L-NAME vs. CON groups, irrespective of sex. **h** Representative short axis M-mode pictures. Each data point represents values derived from individual experimental animals. For statistical analyses, two-way ANOVA with Sidak’s post-hoc test were used. All values are presented as mean ± standard deviation. *: *P* < 0.05 vs. corresponding control. *LVEDD* left ventricular end-diastolic diameter, *LVESD* left ventricular end-systolic diameter, *LVPWTd* left ventricular posterior wall thickness in diastole

Thirteen male and twelve female mice aging 17 months were randomly assigned to receive a control diet (SAFE 210-U8959, SAFE Custom Diets, Augy, France) or high-fat diet (SAFE 210-U8959, SAFE Custom Diets, Augy, France, comprised of 13.0% proteins, 58.6% fats, and 28.4% carbohydrates) plus *Nω*-nitro-L-arginine methyl ester hydrochloride in drinking water (L-NAME, N5751, Merck, Darmstadt, Germany, 0.5 g/L dissolved in drinking water, and pH was adjusted to 7.4), i.e., to CON and HFD + L-NAME study groups, respectively. Diet and water remained to be available freely throughout the study period. After 17 weeks of dietary treatment, echocardiographic analyses were performed to assess cardiac morphology and functions, followed by the collecting tissue samples for further histological and western blot analyses after humane euthanasia by pentobarbital (90 mg/kg, i.p.) (Fig. 1a).

In an additional set of experiments, twelve male and thirteen female 17-month-old mice were randomly assigned to receive CON or HFD + L-NAME diet for a total of 17 weeks, and were treated with anti-PD-1 monoclonal antibody (ICI) (clone RMP1-14, BP0146, BioXCell, Lebanon, NH, USA) intraperitoneally, for 2 weeks (from week 15 to week 17 of the dietary treatment), three times a week, 200 µg ICI dissolved in 200 µL of PBS. We chose 2 weeks of ICI treatment, as (i) in previously published studies, this treatment duration was sufficient to elucidate systolic dysfunction [13, 35] and (ii) we sought to minimize treatment duration for the 21-month-old animals. In this study, echocardiography was performed before ICI initialization (baseline) and at termination, followed by collecting tissue samples for further histological analyses after humane euthanasia (Fig. 4a).

We excluded one animal from the echocardiographic and cardiac histology analyses due to the inability to obtain optimal echocardiographic cines from the female HFD + L-NAME group.

### Echocardiography

Echocardiographic measurements and evaluations were performed as described earlier [11, 40, 42]. Briefly, mice were anesthetized with a mixture of isoflurane and oxygen (5 V/V% for induction, 2 V/V% for maintenance), body temperature was maintained at 37 ± 0.5 °C by a heating pad, and ECG was recorded throughout the measurements. Cardiac functions were assessed with the Vevo 3100 high-resolution in vivo imaging system (Fujifilm VisualSonics, Toronto, Canada) with an MX400 transducer. Two-dimensional recordings of the heart were obtained by long-axis view for volumetric analyses, and short-axis views with M-mode for ventricular diameter and wall thickness analyses. Apical four-chamber view was used to measure early mitral inflow velocity (*E*) with pulse-wave Doppler, and mitral annular early diastolic velocity (*e*’) with tissue Doppler, and the ratio of *E*/*e*’ was calculated.

Stroke volume (LVSV) was calculated as left ventricular (LV) end-diastolic volume (LVEDV) minus LV end-systolic volume (LVESV). Ejection fraction (EF) was calculated as [LVSV/LVEDV × 100]. Cardiac output (CO) was determined as SV × HR/1000. LV diameter during systole and diastole (LVESD and LVEDD, respectively), LV anterior and posterior wall thickness in both systole and diastole (LVAWTs, LVPWTs, LVAWTd, and LVPWTd, respectively) were measured. Fractional shortening (FS) was calculated with the following formula: [(LVEDD − LVESD)/LVEDD] × 100. LV mass was calculated as {[(LVEDD + LVAWTd + LVPWTd)^3^–LVEDD^3^] × 1.0} × 0.8 + 0.14. LV remodeling index (LVRi) was calculated as (LVmass/LVEDD).

Echocardiographic measurements were performed by an operator blinded to the study groups. Echocardiographic recordings were evaluated with the VevoLAB software by an operator blinded to the study groups.

### Histological analysis

Histological analyses were performed as described earlier [26, 27]. Briefly, cardiac samples were fixed in 10% neutral buffered formalin for 24 h, then dehydrated and embedded in paraffin. Five µm thick sections were cut with a microtome. All staining was visualized and captured with a Leica LMD6 microscope (Wetzlar, Germany). For assessing cardiac fibrosis, cardiac sections were stained with 0.0125% picrosirius-red for 1 h, and then washed with 1% acetic acid. Quantification of fibrosis was assessed by ImageJ software. To assess cardiomyocyte hypertrophy and microvascular density, cardiac sections underwent antigen retrieval (citrate buffer pH 6, Vector Laboratories, Newark, CA, USA, H-3300) for 30 min, then incubated with wheat germ agglutinin (WGA–FITC—marker of cell membrane, 1:50, Sigma Aldrich, L4895) and with isolectin B4 (ILB4-DyLight 594—marker of cardiac endothelial cells, 1:50, Invitrogen, L32473) overnight at 4 °C. Five images were captured from the endocardial region of each heart sample. The cardiomyocyte cross-sectional area was automatically segmented with ImageJ Software. Microvascular density was calculated as the ratio of capillary count and the average of cardiomyocyte cross-sectional area. All histologic procedures and analyses were performed by operators blinded to the study groups.

### Western Blot

Frozen spleen samples were homogenized in RIPA lysis buffer (20 mM of Tris-HCl, 150 mM of NaCl, 1% NP-40). Protein concentration was measured by the bicinchoninic acid method using bovine serum albumin (BSA) as standard (Thermo Fisher Scientific, Rockford, IL, United States). Twenty-five μg of protein was loaded onto 4–20% polyacrylamide gel. After separation by gel electrophoresis, proteins were transferred (Criterion Blotter, BioRad, Hercules, CA, United States) onto PVDF membranes (BioRad, Hercules, CA, United States). After successful transfer, membranes were blocked with 5% BSA (Sigma-Aldrich, St. Louis, MO, United States) solution for 1 h at room temperature. Afterward, the membrane was incubated overnight at 4 °C with the following primary antibodies (dissolved in 5% BSA solution) against Tox/Tox2 (E6G5O, Cell Signaling Technology, Danvers, MA, United States, 1:1,000 dilution), TCF1/7 (C63D9, Cell Signaling Technology, Danvers, MA, United States, 1:1,000 dilution), PD-1 (D7D5W, Cell Signaling Technology, Danvers, MA, United States, 1:1,000 dilution), TIM3 (ab241332, Abcam, Cambridge, MA, United Kingdom, 1:1,000 dilution), VISTA (D5L5T, Cell Signaling Technology, Danvers, MA, United States, 1:1,000 dilution), and OX40 (E9U7O, Cell Signaling Technology, Danvers, MA, United States, 1:1,000 dilution) followed by washing with 0.05% Tris-buffered saline with Tween 20 (TBS-T) (3 × 10 min). The membrane was incubated with a secondary antibody (horseradish peroxidase-conjugated goat anti-rabbit, 7074, Cell Signaling Technology, Danvers, MA, United States, 1:5,000 dilution) dissolved in 5% BSA solution for 2 h at room temperature, followed by 3 × 10 min wash. For band detection, the membranes were incubated with enhanced chemiluminescence reagent (Clarity Max Western, BioRad, Hercules, CA, United States) for 5 min and the signal was recorded with the ChemiDoc XRS + System (BioRad, Hercules, CA, United States). Band intensity was evaluated using the Image Lab Software (BioRad, Hercules, CA, United States) and was normalized to total protein content.

### Enzyme-linked immunoassay (ELISA)

For assessing the serum concentration of cholesterol and cardiac Troponin-I, cholesterol ELISA (CHOL-MC-0530, NS BIOTEC, Alexandia, Egypt), and ultrasensitive mouse cardiac Troponin-I ELISA (CTNI-1-US, Life Diagnostics, West Chester, PA, USA) were used according to the manufacturer’s instructions. To evaluate the serum levels of circulating cytokines, BioLegend LEGENDplex multiplex bead-based sandwich ELISA method was performed in accordance with the manufacturer’s instructions. This immunoassay utilizes APC conjugated beads, which are differentiated by their size and internal fluorescence intensities. Each bead set (A5, A8, A10, B2, B4, and B9) is conjugated with a specific antibody on its surface and serves as a capture bead for the analyte. After bead-analyte-detection antibody sandwiches are formed, streptavidin–phycoerythrin (SA–PE) is subsequently added, which binds to the detection antibodies and provides fluorescent signal intensities in proportion to the number of bound analytes. The concentration of a particular analyte is determined using a standard curve generated in the same assay. In our B-cell mix and match panel we measured the following targets on Cytek Northern Lights flow cytometer: IL-2, IL-6, IL-10, TNFα, TGFβ, IFNγ. The concentrations were calculated using LEGENDplex Data Analysis software by Qognit.

### Statistical analysis

All data were generated from at least five independent experiments, i.e., each data point represents values derived from individual experimental animals. All values are presented as mean ± standard deviation (SD). The statistical analysis was performed using GraphPad Prism software (version 8.0.1). *P* < 0.05 was considered significant in each comparison. ROUT analysis was performed to identify outliers, with *Q* value = 1%. Testing for normality of the distribution was performed by the Shapiro–Wilk test. For comparisons between two groups, either a parametric two-tailed Student’s *t* test or a non-parametric Mann–Whitney *U* test was performed (for values of normal or non-normal distribution, respectively). For comparisons between four groups with 2 independent variables (i.e., sex and diet), two-way ANOVA with Sidak’s post-hoc test was performed in case of normal distribution, or Kruskal–Wallis test with Dunn’s post-hoc test was performed in case of non-normal distribution. *P* values for interaction of the two dependent variables (*P*_INT_), or for the separate independent variables (*P*_SEX_—comparing males vs. females, irrespective of the diet; *P*_DIET_—comparing CON vs. HFD + L-NAME, irrespective of sex) were calculated. For the comparison between baseline and termination data, repeated measures two-way ANOVA with Sidak’s post-hoc test was performed.

## Results

### HFD + L-NAME induces left ventricular remodeling in both sexes of aged mice, but diastolic dysfunction only in the male

To assess the effect of prevalent obesity and hypercholesterolemia on the T-cell differentiation states, we fed 17-month-old mice of both sexes with CON or HFD + L-NAME for 17 weeks (Fig. 1a). This dietary regime is a well-established pre-clinical model for cardio-metabolic co-morbidities [47], encompassing obesity, hypercholesterolemia, insulin resistance, hypertension, and diastolic dysfunction, which has never been tested in aged animals. After completion of the dietary period echocardiographic measurements, followed by tissue sample collection were performed.

Before the start of the dietary intervention, male mice had significantly higher body weight compared to females, and there were no significant differences within sexes, as expected (Supplementary Fig. S1a). HFD + L-NAME led to a significant increase in body weight (Fig. 1b) and in heart weight (Fig. 1c), independently of sex (*P*_DIET_ < 0.05 for both analyses). Echocardiographic analyses showed no change in ejection fraction in all study groups (Fig. 1d), whereas significantly increased *E*/*e*’ values were observed in males, but not in females (*P*_DIET_ not significant) (Fig. 1e). Nevertheless, echocardiographic parameters for left ventricular remodeling (i.e., LVAWTd and LVRi) showed a significant increase in HFD + L-NAME vs. CON, independently of sex, in line with the heart weight increase (*P*_DIET_ < 0.05 for both analyses) (Fig. 1f–h).

Overall, as expected, HFD + L-NAME induced a significant increase in body weight, and a significant left ventricular remodeling with preserved ejection fraction in both sexes, however, diastolic dysfunction was observed only in male, but not in female aged mice.

### HFD + L-NAME induces cardiomyocyte hypertrophy and cardiac fibrosis only in male aged mice

**Fig. 2:**
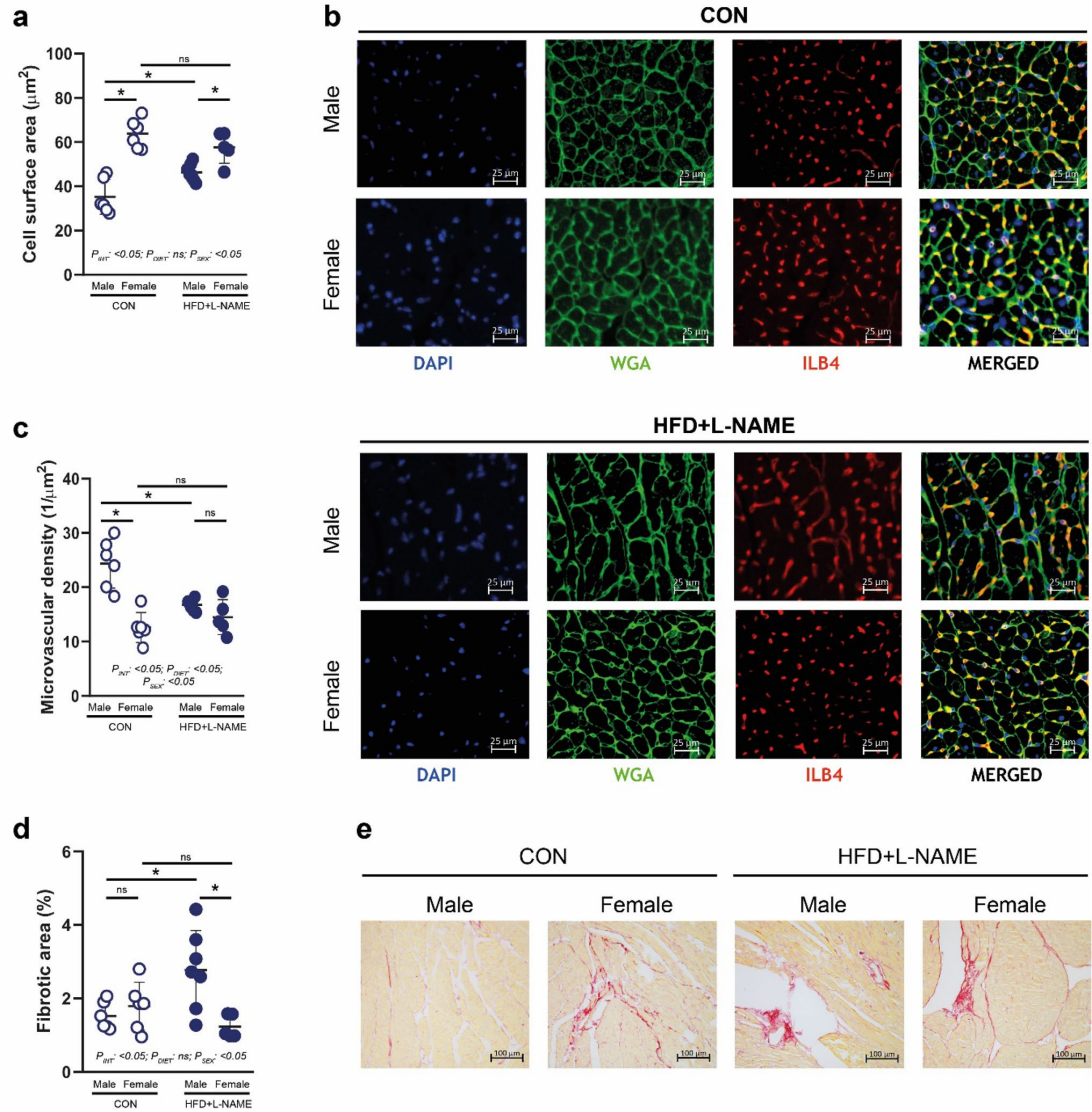
Histological analyses of the hearts. **a** Cell surface area is significantly increased in the male HFD + L-NAME vs. CON diet groups, nevertheless, hypertrophy is present in CON-fed females, and is not further enhanced by HFD + L-NAME. **b** Representative images for cell surface area and microvascular density analyses. **c** Microvascular density is significantly decreased in the male HFD + L-NAME vs. CON diet groups, nevertheless, smaller microvascular density is present in CON-fed females, and is not further decreased by HFD + L-NAME. **d** Fibrotic area is significantly increased in the male HFD + L-NAME vs. CON diet groups, nevertheless, no change in females is achieved. **e** Representative images for fibrotic area analyses. Each data point represents values derived from individual experimental animals. For statistical analyses, two-way ANOVA with Sidak’s post-hoc test were used. All values are presented as mean ± standard deviation. *: *P* < 0.05 vs. corresponding control. *CON* control diet (*n* = 6 for males and *n* = 6 for females), *HFD* + *L-NAME* high fat diet plus L-NAME (HFD + L-NAME, *n* = 7 for males and *n* = 5 for females), *WGA* wheat germ agglutinin, *ILB4* isolectin B4

After the completion of echocardiographic measurements, serum samples were obtained to perform cholesterol level measurements, and cardiac samples were obtained to perform histologic analyses. As expected, HFD + L-NAME led to a significant increase in serum cholesterol levels in both sexes (Supplementary Fig. S2a). HFD + L-NAME led to a significantly increased cardiomyocyte cell surface area in male mice vs. CON. Unexpectedly, female mice showed a significantly higher cell surface area vs. males, irrespective of the diet, and cell surface area was not affected by HFD + L-NAME diet in the female sex (*P*_SEX_ < 0.05) (Fig. 2a, b). Similarly, HFD + L-NAME led to a significantly decreased cardiac microvascular density in male mice vs. CON, but in females, microvascular density was significantly lower than in males, irrespective of the diet (*P*_SEX_ < 0.05). Again, HFD + L-NAME did not affect microvascular density in females (Fig. 2c). HFD + L-NAME led to a significantly increased fibrotic area in the male hearts vs. CON, but showed no effect in females (Fig. 2d, e).

In summary, in line with the echocardiographic findings, histologic analyses showed that HFD + L-NAME led to adverse cardiac remodeling in the aged male mice, but did not affect that of the female counterparts. In addition, in females, cell surface area was significantly higher, and microvascular density was significantly lower vs. males, in both the CON and HFD + L-NAME diets.

### Splenic expression of effector T-cell function markers is affected by the diet and sex in aged mice

To assess whether and how sex and prevalent cardio-metabolic comorbidities affect the expression of markers for T-cell function, splenic samples were taken for western blot analyses. The spleen was chosen for this investigation, as this organ has the greatest reservoir for T-lymphocytes with a direct connection to the systemic circulation.

**Fig. 3:**
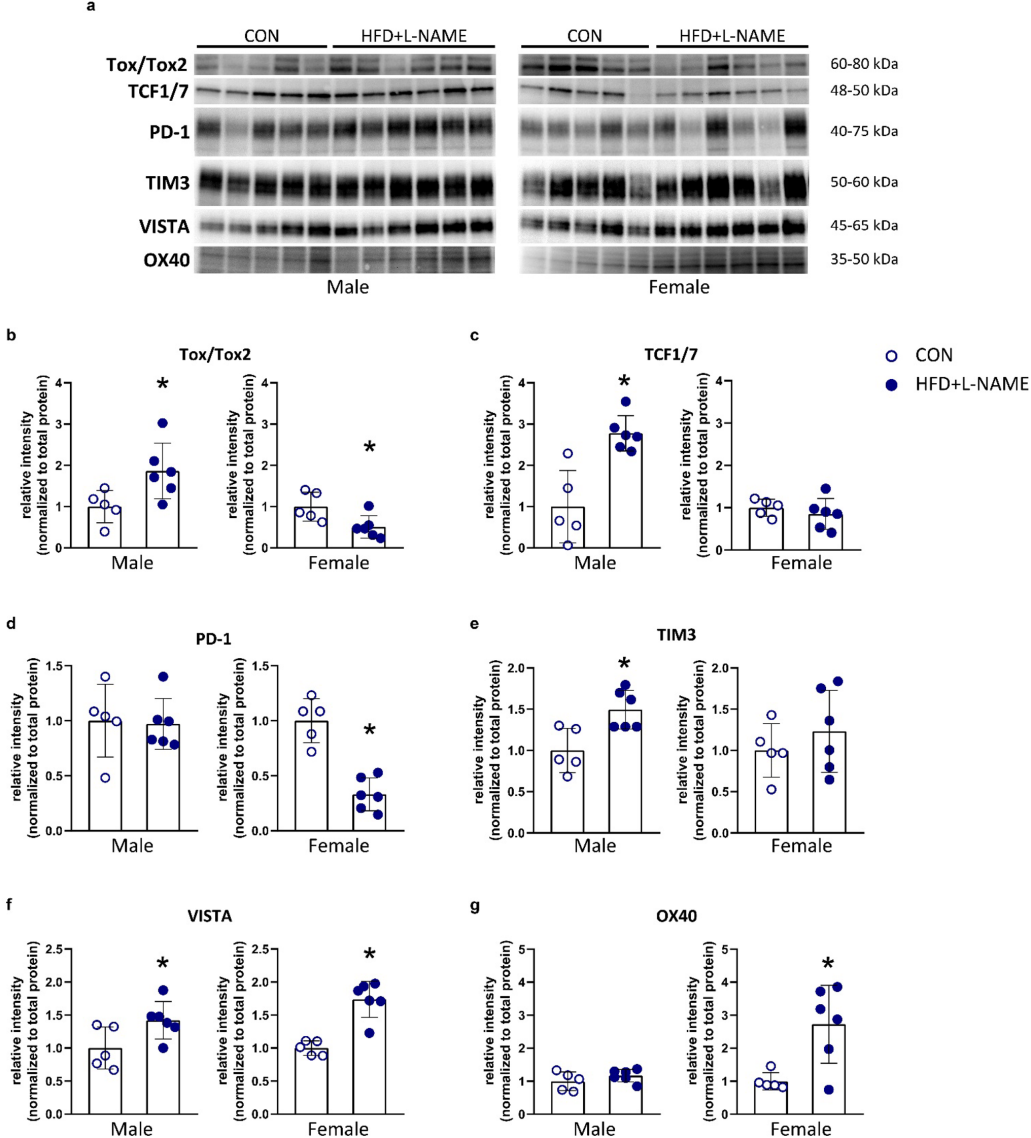
Western blot analyses of spleens. **a** Representative western blot pictures for all analyzed proteins. **b** Splenic protein expression of Tox/Tox2 in males and females. **c** Splenic protein expression of Tcf1/7 in males and females. **d** Splenic protein expression of PD-1 in males and females. **e** Splenic protein expression of TIM3 in males and females. **f** Splenic protein expression of VISTA in males and females. **g** Splenic protein expression of OX40 in males and females. For statistical analyses, Student’s unpaired *t*-test was used. All values are presented as mean ± standard deviation. *: *P* < 0.05 vs. corresponding control. *CON* control diet (*n* = 5 for males and *n* = 5 for females), *HFD* + *L-NAME* high fat diet plus L-NAME (HFD + L-NAME, *n* = 6 for males and *n* = 6 for females), *WGA* wheat germ agglutinin, *ILB4* isolectin B4

Tox/Tox2 and TCF1/7, two major transcription factors associated with the transcription of immune checkpoint genes that are induced during T-cell exhaustion [43, 60], were significantly increased in aged male mice on HFD + L-NAME vs. CON. In female mice on HFD + L-NAME, a significant decrease in Tox/Tox2, and no change in TCF1/7 protein expression were observed vs. CON (Fig. 3a–c). PD-1 and TIM-3 are two inhibitory immune checkpoints that show higher expression during T-cell exhaustion [54]. Although in aged males, HFD + L-NAME did not affect PD-1 expression, it led to a significant increase in TIM-3 expression. On the other hand, HFD + L-NAME led to a significant decrease in PD-1, and no change in TIM-3 expression in aged females (Fig. 3a, d, e). HFD + L-NAME led to a significant increase in VISTA in both sexes (Fig. 3a, f). The expression of OX40, a co-stimulatory immune checkpoint [5] was not affected by the diet in males, but was significantly increased in females on HFD + L-NAME vs. CON (Fig. 3a, g). In addition, we also analyzed the levels of circulating cytokines related to T-cell activity (i.e., IL-2, IL-6, IL-10, TNFα, TGFß, and IFNγ), and found no significant diet-related differences in the circulating cytokine levels of both sexes (Supplementary Table S1).

In conclusion, the HFD + L-NAME dietary regime was associated with a splenic expression pattern of T-lymphocyte markers indicative of T-cell exhaustion that was more pronounced in aged males. Based on this, we hypothesized that ICI-related cardiotoxic side effects may also differ in HFD + L-NAME vs. CON, and also, in male vs. female.

### PD-1 inhibition leads to systolic dysfunction in CON, but not in HFD + L-NAME-fed mice

**Fig. 4:**
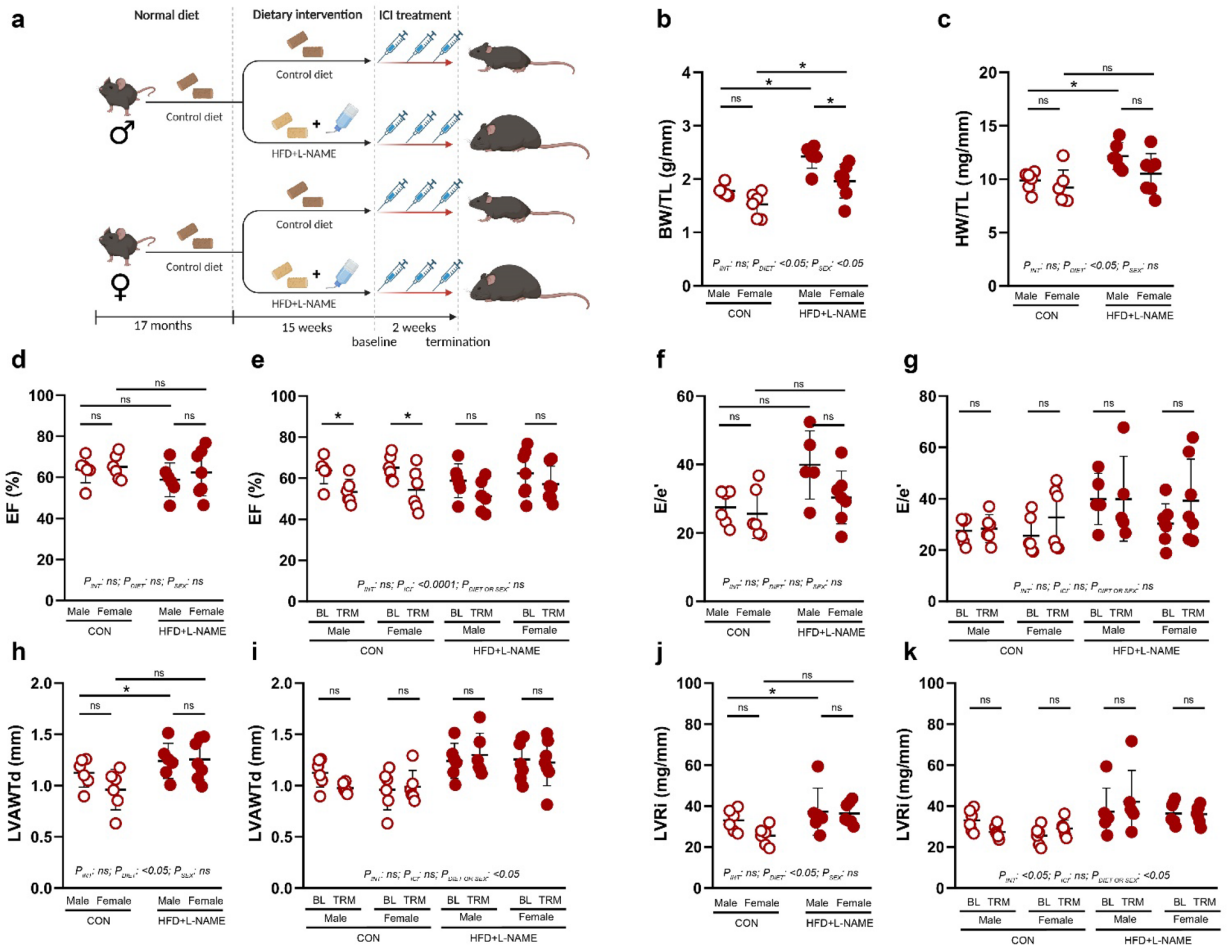
Study desing, gross data and echocardiographic measurements of the effects of immune checkpoint inhibition in aged mice fed with CON vs HFD + L-NAME. **a** Study design depicting that male and female mice of 17 months of age were randomized into control diet (CON, *n* = 6 for males and *n* = 6 for females) or high fat diet plus L-NAME (HFD + L-NAME, *n* = 6 for males and *n* = 7 for females), receiving immune checkpoint inhibitor (ICI) for the last 2 weeks of their dietary intervention. **b** Body weight (BW) normalized to tibia length (TL) is significantly higher in the HFD + L-NAME vs. CON groups, irrespective of sex. **c** Heart weight (HW) normalized to TL is significantly higher in the HFD + L-NAME vs. CON groups, irrespective of sex. **d** Left ventricular ejection fraction (EF) at baseline is not affected by the diet or sex. **e** EF at baseline (BL) vs. termination (TRM) is significantly decreased after ICI treatment in the CON-fed groups, but not in the HFD + L-NAME groups, in both sexes. **f** *E*/*e*’ ratio, a major determinant for diastolic dysfunction is increased in males, but not in females of the HFD + L-NAME-fed groups at baseline. **g** *E*/*e*’ at BL vs. TRM does not change in any of the groups after ICI treatment. **h** Left ventricular anterior wall thickness in diastole (LVAWTd) at baseline is significantly higher in the HFD + L-NAME vs. CON groups, irrespective of sex. **i** LVAWTd at BL vs. TRM does not change in any of the groups after ICI treatment. **j** Left ventricular remodeling index (LVRi) at baseline is significantly higher in the HFD + L-NAME vs. CON groups, irrespective of sex. **k** LVRi at BL vs. TRM does not change in any of the groups after ICI treatment. Each data point represents values derived from individual experimental animals. For statistical analyses, two-way ANOVA with Sidak’s post-hoc test were used for ‘gross data’ and ‘baseline echocardiographic data’, and repeated measures two-way ANOVA with Sidak’s post-hoc test were used for ‘BL vs. TRM’ analyses. All values are presented as mean ± standard deviation. *: *P* < 0.05 vs. corresponding control. *LVEDD* left ventricular end-diastolic diameter, *LVESD* left ventricular end-systolic diameter, *LVPWTd* left ventricular posterior wall thickness in diastole

To test the hypothesis of whether and how ICI-induced cardiotoxic side effects are affected by the diet or sex, we treated aged mice of both sexes either on CON or on HFD + L-NAME diet with the ICI anti-PD-1 monoclonal antibody for 2 weeks, on the last 2 weeks of their diet (Fig. 4a). Echocardiographic measurements were performed before the initiation of ICI (baseline), and at termination.

In accordance with the previous experiment, baseline bodyweights did not differ within sexes before the start of the diet (Supplementary Fig. S1b), and HFD + L-NAME resulted in a significant increase in body weights (Fig. 4b) and heart weights (Fig. 4c) of both sexes in aged mice vs. CON. At baseline, echocardiographic parameters were similar to that of the previous study, i.e., no difference in systolic function was observed between the groups (Fig. 4d), diastolic dysfunction was present only in the male HFD + L-NAME group vs. male CON (Fig. 4f), and evidence for adverse cardiac remodeling was seen in HFD + L-NAME vs. CON, irrespective of sex (Fig. 4h, j).

Two weeks of ICI treatment-induced significant systolic dysfunction in CON mice of both sexes vs. the corresponding baseline, however, interestingly, no such effect was seen in the HFD + L-NAME fed groups (Fig. 4e). ICI treatment did not affect the diastolic function and measures of adverse cardiac remodeling, as no changes in *E*/*e*’ (Fig. 4g), LVAWTd (Fig. 4i), and LVRi (Fig. 4k) were observed in any groups between baseline and termination. In addition, high-sensitivity Troponin-I measurements evidenced a lack of acute myocardial injury for ICI treatment, irrespective of sex and diet (Supplementary Fig. S3).

Overall, 2 weeks of ICI treatment caused systolic dysfunction in CON-fed animals of both sexes, whereas no such effects were seen in HFD + L-NAME groups. This observation was paralleled by no changes in diastolic function or cardiac morphology.

### PD-1 inhibition does not affect cardiomyocyte hypertrophy induced by HFD + L-NAME

**Fig. 5:**
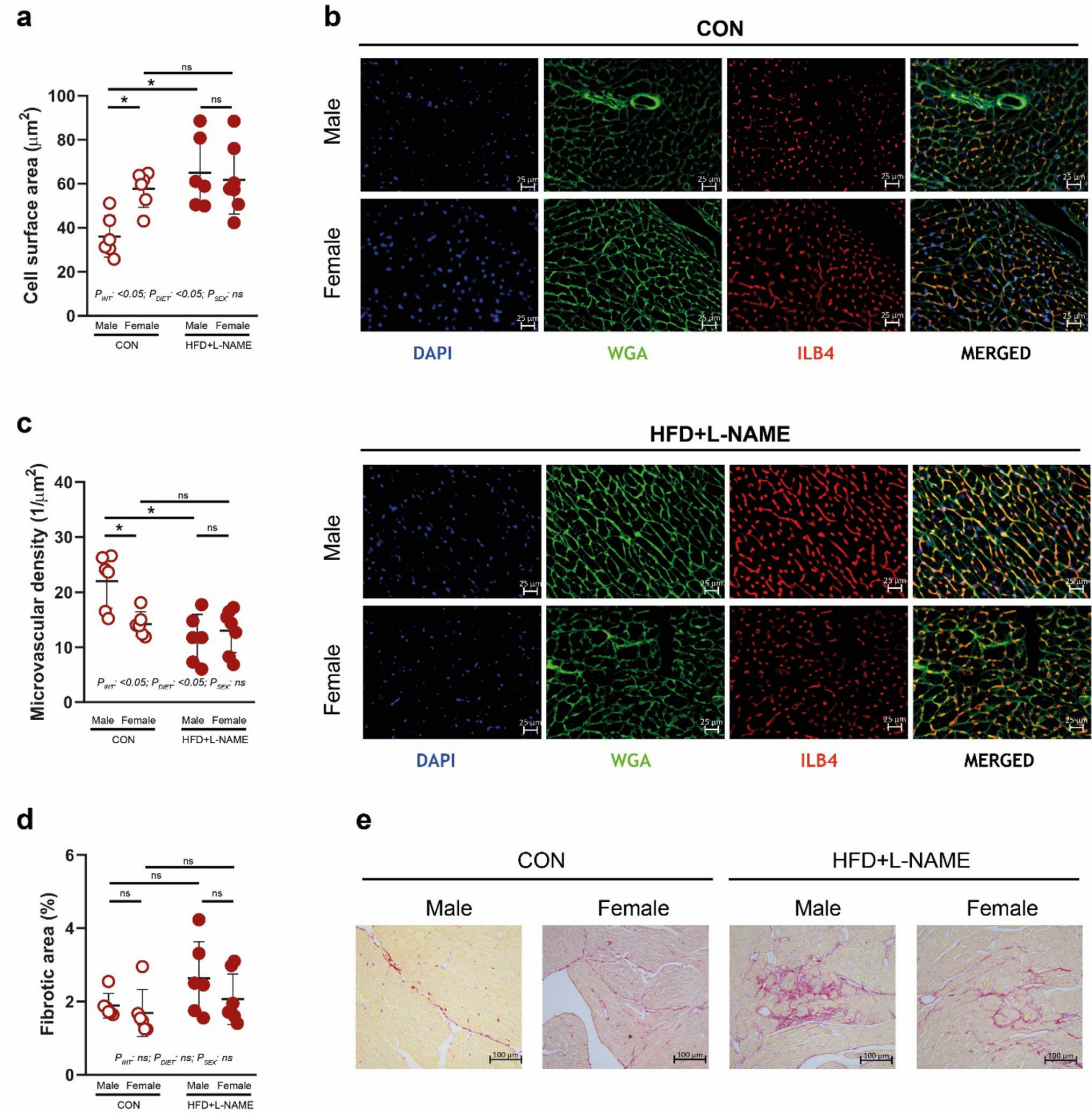
Histological analyses of the hearts of mice treated with immune checkpoint inhibitor. **a** Cell surface area is significantly increased in the male HFD + L-NAME vs. CON diet groups, nevertheless, hypertrophy is present in CON-fed females, and is not further enhanced by HFD + L-NAME. **b** Representative images for cell surface area and microvascular density analyses. **c** Microvascular density is significantly decreased in the male HFD + L-NAME vs. CON diet groups, nevertheless, smaller microvascular density is present in CON-fed females, and is not further decreased by HFD + L-NAME. **d** Fibrotic area does not change in any of the groups irrespective of sex or diet. **e** Representative images for fibrotic area analyses. Each data point represents values derived from individual experimental animals. For statistical analyses, two-way ANOVA with Sidak’s post-hoc test were used. All values are presented as mean ± standard deviation. *: *P* < 0.05 vs. corresponding control. *CON* control diet (*n* = 6 for males and *n* = 6 for females), *HFD* + *L-NAME* high fat diet plus L-NAME (HFD + L-NAME, *n* = 6 for males and *n* = 7 for females), *WGA* wheat germ agglutinin, *ILB4* isolectin B4

After the completion of echocardiographic measurements, serum samples were obtained to perform cholesterol level measurements, and cardiac samples were obtained to perform histologic analyses. Again, HFD + L-NAME led to a significant increase in serum cholesterol levels in both sexes (Supplementary Fig. S2b). As observed in the previous experiment, HFD + L-NAME led to a significantly increased cardiomyocyte cell surface area in male mice vs. CON, but did not affect that of the female mice, and ICI treatment did not cause a change in this relation (Fig. 5a, b). Similarly, HFD + L-NAME led to a significantly decreased cardiac microvascular density in male mice vs. CON, but in females, microvascular density was significantly lower than in males, irrespective of the diet, and again ICI treatment did not interfere with these effects (Fig. 5c). Nevertheless, in ICI-treated mice, no significant difference was observed between the groups regarding cardiac fibrosis, irrespective of sex or diet, as opposed to the previous experiment without ICI treatment.

In summary, ICI treatment did not affect the hypertrophic effect of HFD + L-NAME treatment, as similar patterns were seen in animals without ICI treatment, i.e., HFD + L-NAME led to adverse cardiac remodeling in the aged male mice, but did not affect that of the female counterparts, and that in female mice cell surface area was significantly higher, and microvascular density was significantly lower vs. males of the CON groups.

### PD-1 inhibition changes diet-related differences in splenic expression of effector T-cell function markers in both sexes of aged mice

To see whether the sex- and diet-dependent expression pattern of splenic effector T-cell function markers changes after ICI treatment, we performed a western blot analysis of splenic samples from ICI-treated animals, assessing the same targets as in the previous study.

**Fig. 6:**
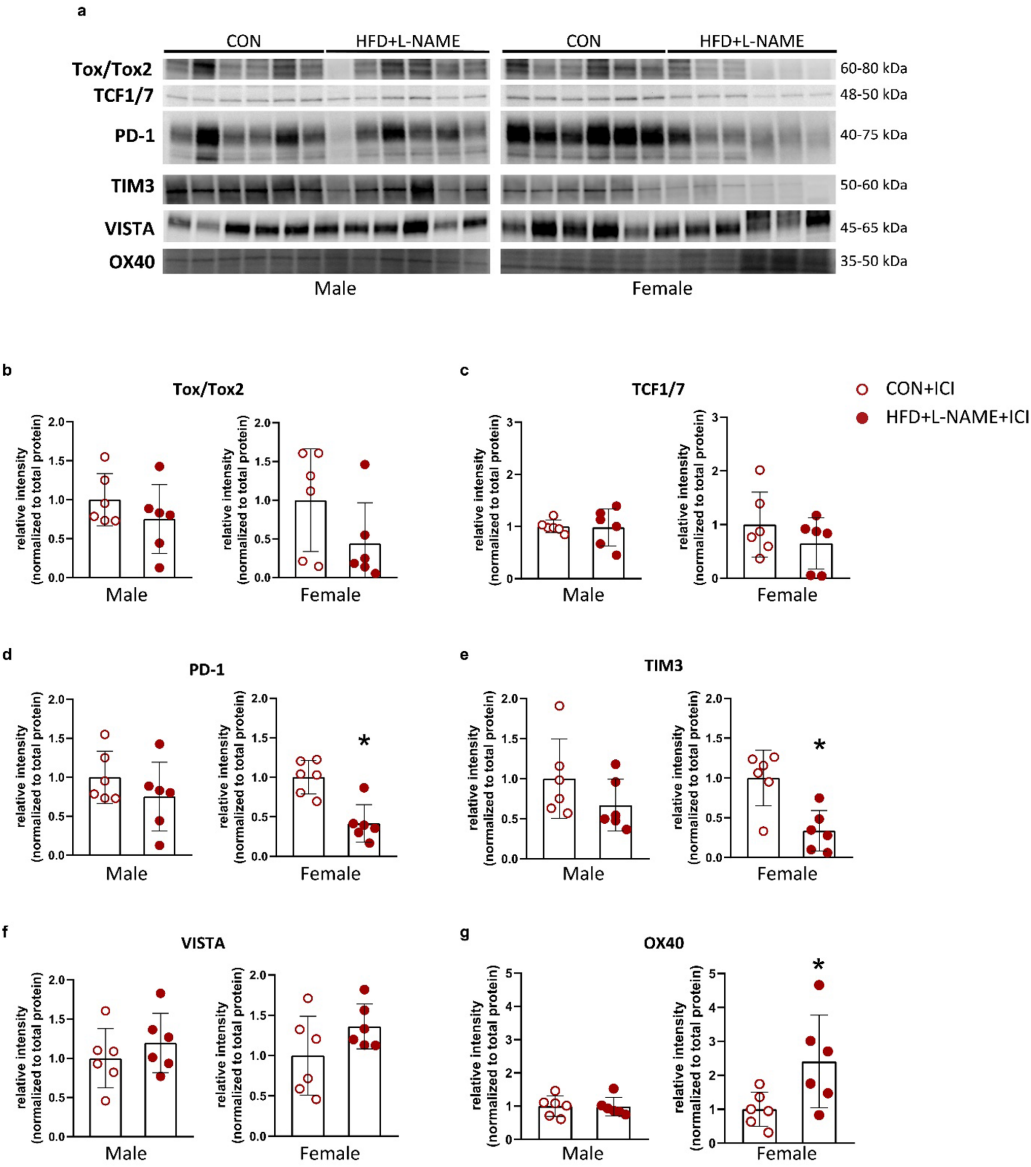
Western blot analyses of spleens of mice treated with immune checkpoint inhibitor. **a** Representative western blot pictures for all analyzed proteins. **b** Splenic protein expression of Tox/Tox2 in males and females. **c** Splenic protein expression of Tcf1/7 in males and females. **d** Splenic protein expression of PD-1 in males and females. **e** Splenic protein expression of TIM3 in males and females. **f** Splenic protein expression of VISTA in males and females. **g** Splenic protein expression of OX40 in males and females. For statistical analyses, Student’s unpaired *t*-test was used. All values are presented as mean ± standard deviation. *: *P* < 0.05 vs. corresponding control. *CON* control diet (*n* = 6 for males and *n* = 6 for females), *HFD* + *L-NAME* high fat diet plus L-NAME (HFD + L-NAME, *n* = 6 for males and *n* = 6 for females), *WGA* wheat germ agglutinin, *ILB4* isolectin B4

We found that in ICI-treated males, the expression of Tox/Tox2, TCF1/7, TIM-3, and VISTA was not anymore significantly increased in HFD + L-NAME vs. CON groups (Fig. 6), in contrast to the previous study with non-ICI-treated animals (Fig. 3). On the other hand, in females, the protein expression of PD-1 remained significantly decreased, and OX40 remained significantly increased, whereas the protein expression of Tox/Tox2 was not anymore significantly decreased in HFD + L-NAME vs. CON groups (Fig. 6), when compared to the non-ICI-treated counterparts (Fig. 3). In addition, the levels of circulating cytokines related to different T-cell functions were not significantly affected by the diet or the sex in ICI-treated animals (Supplementary Table S2), a finding that is similar to that of the non-ICI-treated mice.

Overall, after ICI treatment, diet-related differences in the expression of splenic T-cell function markers of aged mice disappeared in males and became less pronounced in females.

## Discussion

**Fig. 7:**
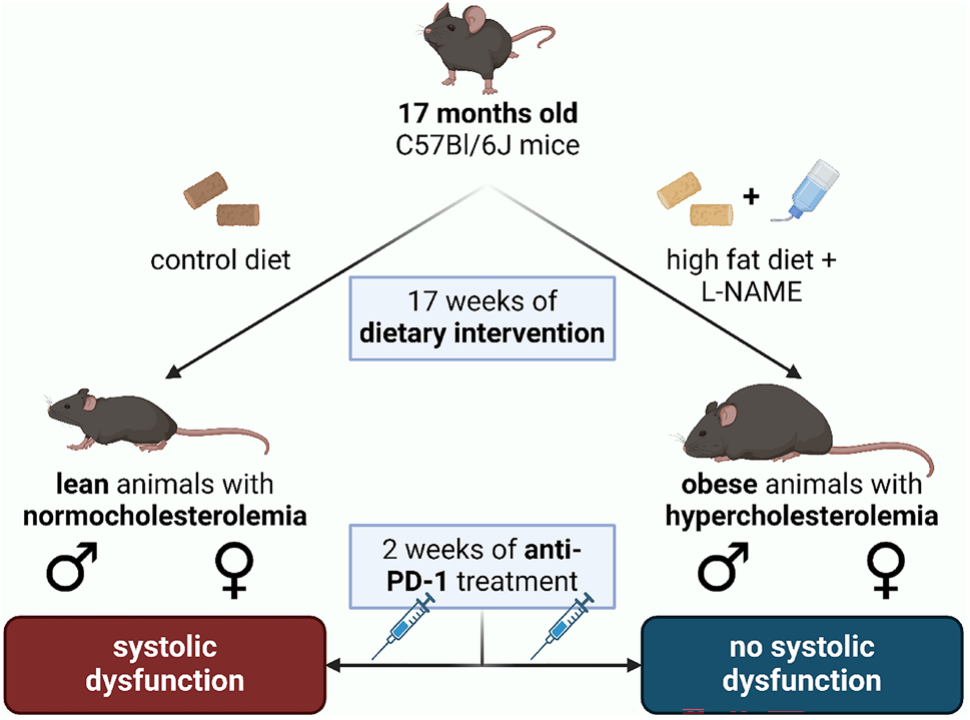
The current study aimed to assess the effects of sex and obesity on immune checkpoint inhibition-related cardiac systolic dysfunction in aged mice. Anti-PD-1 treatment caused systolic dysfunction in the lean, normocholesterolemic mice, nevertheless, systolic dysfunction was not observed in the obese, hypercholesterolemic mice. This effect was independent of the sexes

In this exploratory study, we assessed, for the first time, the effect of sex and prevalent cardio-metabolic conditions on ICI-induced cardiac systolic dysfunction in an aging mouse model, and found that PD-1 inhibition causes significant deterioration in left ventricular ejection fraction only in lean, but not in obese mice, independently of sex (Fig. 7). Here we demonstrated that HFD + L-NAME leads to a phenotype of systemic T-cell exhaustion, which may be associated with lesser effector T-cell activity, and thus, with a diminished cardiotoxicity induced by ICI.

We chose to investigate systolic dysfunction, as a cardiac side effect caused by ICI treatment in the context of obesity and hypercholesterolemia in aging mice, as (i) no myocarditis or cardiac arrhythmias were achieved by the same ICI treatment regime in our previous experiment [13], and (ii) investigations of determinants for cardiotoxicity other than myocarditis are scarce.

As the effect of prevalent cardio-metabolic conditions on ICI-induced cardiac side effects is poorly investigated and understood, we aimed to determine whether and how ICI-induced systolic dysfunction, an increasingly recognized form of ICI-related cardiotoxicity other than myocarditis, is affected by prevalent obesity and hypercholesterolemia. To this end, we used a well-established HFD + L-NAME model in both sexes [47]. With this diet, obesity, dyslipidemia, insulin resistance, hypertension, as well as diastolic dysfunction without alteration in systolic function (i.e., heart failure with preserved ejection fraction) occurs, which was described to be more pronounced in males than in females. Nevertheless, to the best of our knowledge, our study is the first to use this model in aging mice, enhancing clinical relevance, as heart failure with preserved ejection fraction affects the elderly.

Surprisingly, we found for the first time that HFD + L-NAME led to diastolic dysfunction—determined by the strongest echocardiographic parameter, *E*/*e*’[37]—in males, but not in females of this age. However, echocardiographic parameters for adverse cardiac remodeling occurred independently of sex, but to a lesser degree in females. This could be attributed to the fact that cardiomyocyte hypertrophy was prevalent in lean females, which could not be enhanced further by the HFD + L-NAME diet. In addition, fibrosis also occurred to a lesser degree in aged female hearts than in male counterparts. This may be attributed to the fact that the female sex is protective against cardiac hypertrophy and fibrosis due to the effects of estrogen in the pre-menopausal age, however, this protection is abolished after menopause [56]. In the current study, mice of 17 months of age were used, characterized as post-menopausal state, which may explain the above observations.

The model of HFD + L-NAME applied for 5 weeks in young mice has been demonstrated to be associated with an enhanced systemic inflammatory state and has been causally linked to cardiac dysfunction by causing T-cell infiltration and inflammation in the cardiac tissue [45]. In the current study, the HFD + L-NAME diet was applied for 17 weeks in aged mice, and led to systemic T-cell exhaustion, as determined by splenic western blot measurements of immune checkpoint protein levels, which was more pronounced in males than in females. This may be due to the fact that T-cell exhaustion occurs by chronic inflammation.

In the current study, the spleen was chosen as the organ of interest for analysis of T-cell exhaustion markers, as previous studies demonstrated that exhausted T cells accumulate in the red pulp of the spleen [1]. In males, we demonstrated (i) a significant increase in Tox/Tox2 and Tcf1/7, transcription factors responsible for the expression of mostly co-inhibitory immune checkpoint molecules in T cells [43, 60], (ii) a significant increase in TIM3, a co-inhibitory immune checkpoint molecule [54], and (iii) no change in the expression of the co-stimulatory immune checkpoint OX40 [5]. On the other hand, females showed a significant decrease in Tox/Tox2 and in the co-inhibitory PD-1, no change in Tcf1/7 and in TIM3, and a significant increase in OX40. Of interest, previous investigations have shown that the presence of androgens promotes an exhausted phenotype of T cells [28], supporting our findings.

Interestingly, significantly increased protein expression of VISTA was seen in HFD + L-NAME groups of both sexes. VISTA has been linked to the inhibition of T-cell activation and cytokine production, as well as to the reprogramming of myeloid cells for a reduced production of pro-inflammatory cytokines [58]. Although, classical markers for T-cell exhaustion were present in males, and absent in females, the fact that VISTA was increased by HFD + L-NAME in both sexes may explain why fewer cardiac side effects of ICI treatment were seen in both sexes, and not only in males.

Obesity has been linked to T-cell exhaustion in a landmark study by Ziming and colleagues [51]. In this study, obesity led to an increased immune aging and enhanced tumor progression by causing T-cell exhaustion through leptin, but paradoxically, obesity enhanced anti-cancer response and improved overall survival of cancer-bearing mice and cancer patients treated with ICIs. Later, Kichenedasse et al. also demonstrated that obese vs. non-obese patients with non-small cell lung cancer treated with ICI have an improved survival [22]. This ‘obesity-paradox’ in oncology is well-recognized, however, is still debated. Clinically, a large body of observational evidence has linked higher body-mass-index with improved survival of cancer patients, nevertheless, the majority of these studies were subject to criticism due to different forms of biases, uncontrolled confounding factors and reverse causality [30]. From the mechanistic point of view, significant weight loss during cancer treatment and cancer cachexia are associated with poor survival, a factor that has been increasingly recognized recently [29]. Nevertheless, even if obesity would undisputedly improve cancer survival, it should be noted that obesity per se is a risk factor for cancer incidence, i.e., obesity is a double-edged sword.

Obesity-paradox in the field of cardiology has long been a subject of debate, as it is strongly linked to an increased risk of cardiovascular events, mainly driven by atherosclerosis [16], nevertheless, survival benefits of obese patients are reported in a large array of cardiovascular diseases ranging from chronic heart failure to atrial fibrillation [6].

Obesity in anthracycline and trastuzumab-related cardiotoxicity studies has been linked to an increased risk of cardiac side effects [21]. Nevertheless, in observational studies of ICI-related cardiotoxicity, obesity and hypercholesterolemia have been shown to be protective, however, these results should be proven mechanistically in the future [3, 36]. Based on the above considerations, and that of the current study, it is suggested that obesity may be beneficial in cancer patients receiving ICI, as not only the anti-tumor response increases, but also cardiotoxicity diminishes.

However, these results should be carefully interpreted, as (i) obesity has been strongly linked to an increased risk of cardiovascular events, mainly driven by atherosclerosis [16], and also, as (ii) ICIs have been shown to increase atherosclerotic burden [8], potentially contributing to competing risks. In other words, although cancer-related outcomes may be improved, other causes of death (most likely cardiovascular) may dominate. Moreover, in patients with ICI-related myocarditis, obesity is significantly more prevalent than in those without myocarditis [32], further complicating the relationship between obesity and ICI. In addition, in ICI-treated patients without immune-related adverse events, overall survival is significantly lower than in those who developed such side effects of ICIs [50], suggesting a more pronounced T-cell activity, and thus, a higher anti-tumor efficacy.

As for the sex-related differences for ICI-induced immune-related adverse events, clinical data is conflicting, with analyses showing either a higher risk for ICI side effects are observed in women [9, 49], or no differences between sexes in this regard [20]. In the current study, no difference was observed between aged male and aged female mice regarding ICI-related systolic dysfunction. However, in a comprehensive study by Zhang and colleagues, female mice were more susceptible to ICI-induced myocarditis in a sex-hormone-dependent manner [61]. Nevertheless, this result was obtained from young mice, in contrast to our current study, where aged mice were used.

Overall, currently, the onset of ICI-related cardiotoxic side effects cannot be predicted, as studies assessing determinants for these events are either lacking or non-unanimous. Nevertheless, efforts still should be made to either prevent or treat ICI-related cardiotoxicities, which requires a deeper understanding of the pathomechanisms. Previously, our research group identified IL-17a as a key driver and therapeutic target for ICI-related cardiotoxicity [13]. In addition, Michel and colleagues have demonstrated that TNFα-blockade not only reduces cardiac systolic dysfunction caused by anti-PD-1, but does not disrupt the anti-tumor efficacy of ICI [35]. Several mechanistic studies identified endothelial damage as a key driver and mediator of ICI-induced cardiotoxicity. For instance, Efentakis et al. demonstrated that the ICI pembrolizumab exacerbates coronary microvascular endothelial activation and triggers cardiac inflammation, which could be prevented by a high dose of atorvastatin in vivo [10]. Of interest, coronary microvascular dysfunction, and a consequent alteration in the coronary blood flow is present in heart failure with preserved ejection fraction, nevertheless, it is still of debate whether coronary microvascular dysfunction is a cause, consequence, or a bystander in the pathophysiology of this disease [17]. Besides the involvement of T cells and endothelial cells in ICI-related systolic dysfunction, macrophages have also been shown to play a pivotal role in this pathomechanism through M1-polarization [57]. It also should be noted, that different anticancer therapies, e.g., anthracyclines may modify ICI-related signaling in the heart, further complicating the picture [25]. In addition to the immune system-level approach for identifying targets to diminish ICI-related cardiotoxicity, cardioprotective interventions, such as cardiovascular exercise [52, 53] and ischemic conditioning [23, 34] should also be investigated in the future [18], accounting for the fact that aging, hypercholesterolemia and sex strongly influence the efficacy of any currently known cardioprotective approach [2]. Nevertheless, from a clinical perspective, screening and integrated care by multidisciplinary cardio-oncology teams is one of the most effective options for treating these side effects in the clinical setting [14, 48].

In conclusion, in the current exploratory study, we found that ICI-induced cardiac systolic dysfunction is not affected by sex, but is diminished by the prevalence of obesity and hypercholesterolemia. We emphasize that future experimental and clinical studies should focus on identifying determinants for ICI-related cardiac adverse events by accounting for age, sex, cardio-metabolic co-morbidities, and co-medications, and in parallel, future studies should address underlying mechanisms, a strategy that is not restricted to the field of cardio-oncology only [39, 41].

### Limitations

A major limitation of the current study is that the effects of prevalent cardio-metabolic conditions on ICI-related cardiotoxicity were investigated in the absence of cancer. Therefore, our findings should also be interpreted knowing that cancer is a well-known perturbator of immune (and especially T-cell) functions, as well as cardio-metabolic conditions, strongly influencing cardiotoxic and anti-cancer effects of ICI [4, 19, 24, 33, 38, 55]. The complex interrelation between obesity, T-cell function, and cancer should also be investigated in future studies on ICI-related cardiotoxicity, and especially on ICI-related cardiotoxicity other than myocarditis (e.g., atherosclerosis or non-inflammatory systolic dysfunction).

Another limitation is that, for the ICI treatment study, only baseline vs. termination data were obtained, and no parallel isotype-control groups were used, as with this approach, the possible underlying mechanisms could have been explored in a more nuanced manner, as not only the echocardiographic data could have proven cardiotoxicity, but also the histology and Western Blot data. Nevertheless, the primary research question of the current study was whether and how ICI-related systolic dysfunction is affected by obesity and sex. The presence or absence of ICI-related systolic dysfunction was proven by the baseline (before ICI treatment) vs. termination (after 2 weeks of ICI treatment) echocardiographic measurements—a result that is similar to that of our previously published study [13].

## Conclusion and translational impact

Here, we assessed for the first time the effect of sex and prevalent obesity on immune checkpoint inhibition-induced cardiac systolic dysfunction in aged mice. We found that (i) dietary intervention by high-fat diet plus L-NAME leads to diastolic dysfunction only in aged males, alongside with preserved ejection fraction and echocardiographic histologic markers of adverse cardiac remodeling, (ii) obesity is associated with markers of systemic T-cell exhaustion in a sexually determined pattern, which (iii) may prevent immune checkpoint inhibition-induced cardiac systolic dysfunction in both sexes. Nevertheless, these exploratory findings should be supported by future preclinical and clinical studies, as currently, such investigations are either lacking or conflicting.

## Supplementary Information

Below is the link to the electronic supplementary material.

Supplementary file1 (DOCX 705 KB)

## Data Availability

The data are available from the corresponding author on reasonable request.
