# Supplementary material for: Effects of sex and obesity on immune checkpoint inhibition-related cardiac systolic dysfunction in aged mice
Source: Basic Res Cardiol. 2024 Nov 8;120(1):207–23. doi: 10.1007/s00395-024-01088-4 (PMC11790738; doi:10.1007/s00395-024-01088-4)
Supplement: Supplementary file 1 — Supplementary file1 (DOCX 705 KB) [file 395_2024_1088_MOESM1_ESM.docx]

**Supplementary Fig. S1**


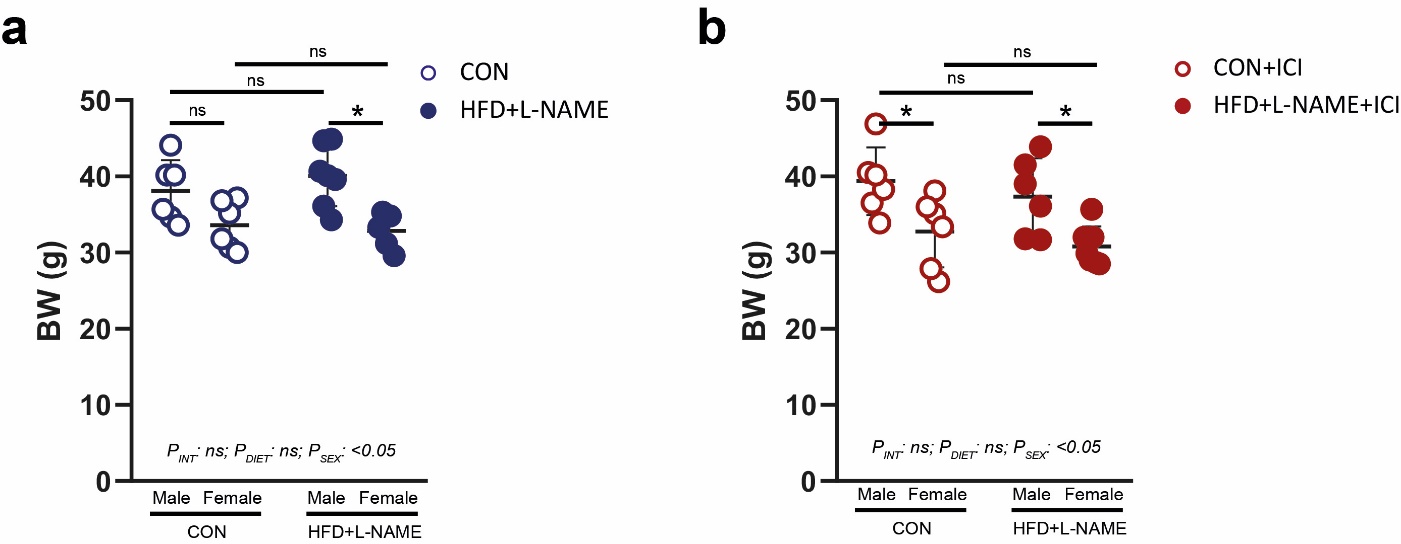


**Supplementary Fig. S1: Baseline (before the start of dietary intervention) body weights of animals.** **a:** animals not treated with ICI. **b:** animals treated with ICI. Each data point represents values derived from individual experimental animals. For statistical analyses, two-way ANOVA with Sidak’s post-hoc test were used. All values are presented as mean±standard deviation. *: p<0.05 vs. corresponding control. CON: control diet, HFD+L-NAME: high fat diet plus L-NAME. BW: body weight.

**Supplementary Fig. S2**

**
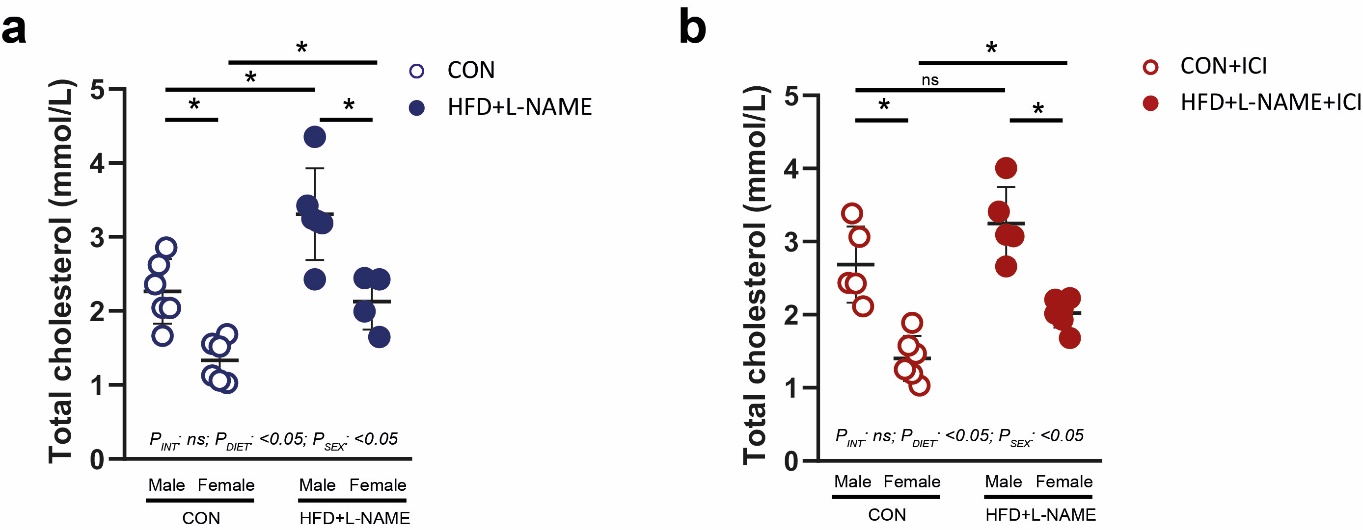
**

**Supplementary Fig. S2: Total cholesterol levels of animals.** **a:** animals not treated with ICI. **b:** animals treated with ICI. Each data point represents values derived from individual experimental animals. For statistical analyses, two-way ANOVA with Sidak’s post-hoc test were used. All values are presented as mean±standard deviation. *: p<0.05 vs. corresponding control. CON: control diet, HFD+L-NAME: high fat diet plus L-NAME.

**Supplementary Fig. S3**

**
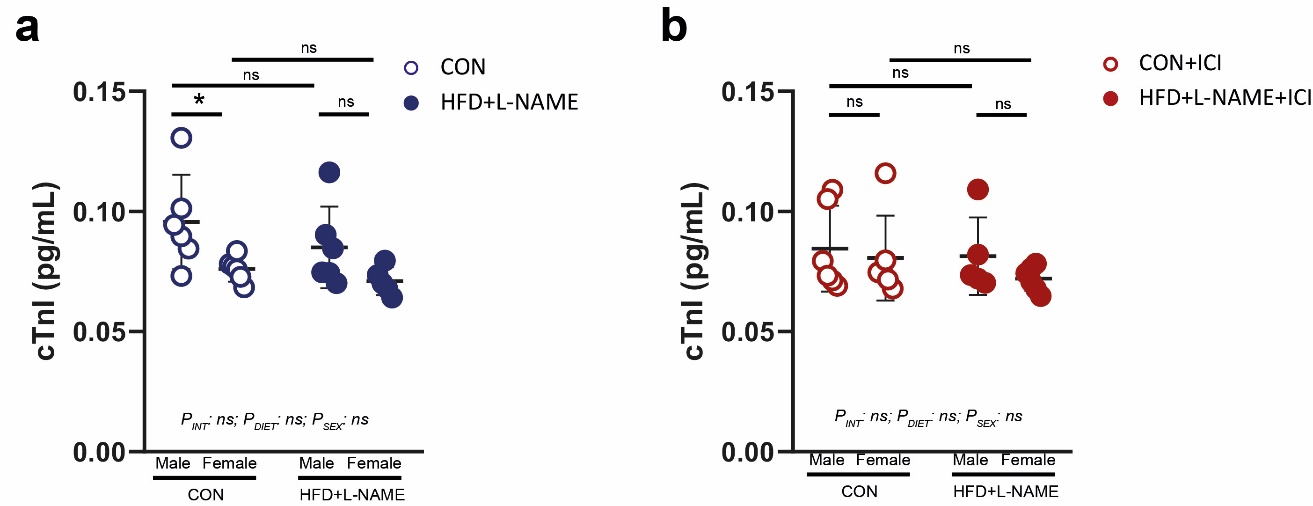
**

**Supplementary Fig. S3: Cardiac Troponin I levels of animals.** **a:** animals not treated with ICI. **b:** animals treated with ICI. Each data point represents values derived from individual experimental animals. For statistical analyses, two-way ANOVA with Sidak’s post-hoc test were used. All values are presented as mean±standard deviation. *: p<0.05 vs. corresponding control. CON: control diet, HFD+L-NAME: high fat diet plus L-NAME. cTnI: cardiac Troponin I.

**Supplementary Fig. S4**

**
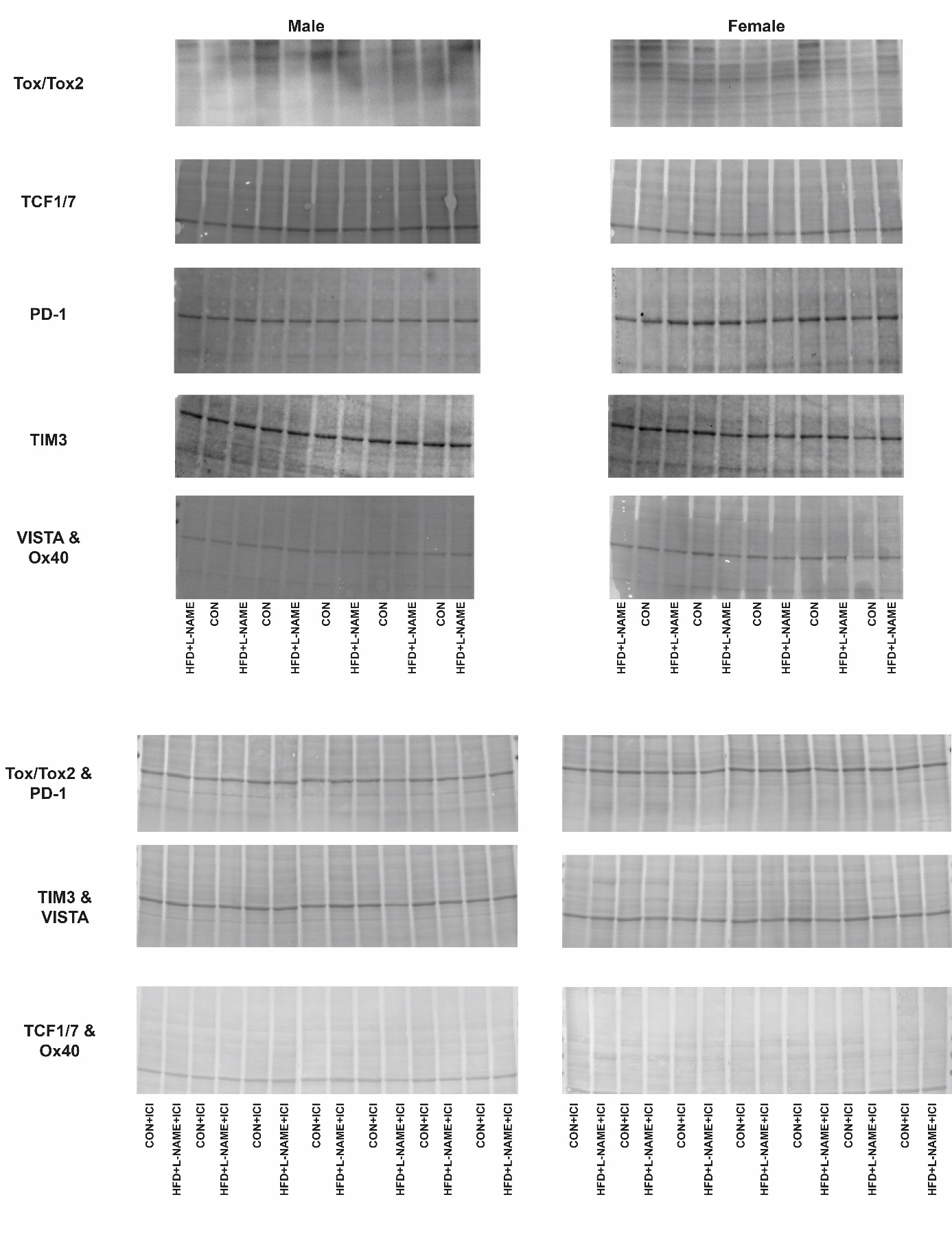
**

**Supplementary Fig. S3: Representative images of the total protein staining for Western Blot.** CON: control diet, HFD+L-NAME: high fat diet plus L-NAME, ICI: immune checkpoint inhibitor.

**Suplementary table S1**

|  | **CON** | | **HFD+L-NAME** | |  |  |  |
| --- | --- | --- | --- | --- | --- | --- | --- |
|  | **Male** | **Female** | **Male** | **Female** | **P_INT_** | **P_SEX_** | **P_DIET_** |
| **IL-2** | 10.35 ± 14.74 | 0.46 ± 1.13 | 9.21 ± 8.76 | 1.19 ± 2.37 | ns | <0.05 | ns |
| **IL-6** | 21.99 ± 26.39 | 5.78 ± 6.85 | 40.14 ± 44.57 | 19.09 ± 25.02 | ns | ns | ns |
| **IL-10** | 6.40 ± 7.14 | 43.98 ± 57.43 | 17.70 ± 15.00 | 5.17 ± 4.59 | ns | ns | ns |
| **TGFß** | 142.6 ± 44.03 | 51.02 ± 34.23 | 66.92 ± 14.85 | 86.96 ± 66.08 | <0.05 | <0.05 | ns |
| **TNFα** | 5.72 ± 5.06 | 5.91 ± 4.60 | 6.61 ± 5.45 | 2.78 ± 1.33 | ns | ns | ns |
| **IFNγ** | 0.30 ± 0.40 | 0.09 ± 0.23 | 0.32 ± 0.42 | 0.43 ± 0.97 | ns | ns | ns |

**Supplementary table S1: Serum cytokine levels of animals not treated with ICI.** For statistical analyses, Kruskal-Wallis test with Dunn’s post-hoc test were used. All values are presented as mean±standard deviation. *: p<0.05 vs. corresponding control. CON: control diet, HFD+L-NAME: high fat diet plus L-NAME.

**Suplementary table S2**

|  | **CON** | | **HFD+L-NAME** | |  |  |  |
| --- | --- | --- | --- | --- | --- | --- | --- |
|  | **Male** | **Female** | **Male** | **Female** | **P_INT_** | **P_SEX_** | **P_DIET_** |
| **IL-2** | 3.65 ± 8.17 | 1.09 ± 2.67 | 4.26 ± 9.78 | 243.2 ± 588 | ns | ns | ns |
| **IL-6** | 6.18 ± 9.39 | 1.74 ± 2.71 | 32.19 ± 37.01 | 101.4 ± 127.7 | ns | ns | ns |
| **IL-10** | 87.42 ± 149.5 | 23.6 ± 33.62 | 10.93 ± 12.18 | 46.92 ± 59.35 | ns | ns | ns |
| **TGFß** | 95.69 ± 52.55 | 62.67 ± 29.36 | 37.73 ± 42.24 | 73.94 ± 83.08 | ns | ns | ns |
| **TNFα** | 6.73 ± 7.75 | 3.29 ± 3.18 | 12.54 ± 14.17 | 7.61 ± 8.22 | ns | ns | ns |
| **IFNγ** | 2.66 ± 5.94 | 0.09 ± 0.23 | 0.53 ± 0.73 | 0.62 ± 0.79 | ns | ns | ns |

**Supplementary table S2: Serum cytokine levels of animals treated with ICI.** For statistical analyses, Kruskal-Wallis test with Dunn’s post-hoc test were used. All values are presented as mean±standard deviation. *: p<0.05 vs. corresponding control. CON: control diet, HFD+L-NAME: high fat diet plus L-NAME.
